# Efficacy of Smear Layer Removal at the Apical One-Third of the Root Using Different Protocols of Erbium-Doped Yttrium Aluminium Garnet (Er:YAG) Laser

**DOI:** 10.3390/medicina59030433

**Published:** 2023-02-22

**Authors:** Amel Yousif Habshi, Nausheen Aga, Khadija Yousif Habshi, Muna Eisa Mohamed Hassan, Ziaullah Choudhry, Muhammad Adeel Ahmed, Azeem Ul Yaqin Syed, Rizwan Jouhar

**Affiliations:** 1 Emirates Health Service, Ministry of Health and Prevention, Sharjah P.O. Box 3241, United Arab Emirates; 2 School of Dentistry, University of Dundee, Dundee DD1 4HN, Scotland, UK; 3 Department of Preventive and Restorative Dentistry, University of Sharjah, Sharjah P.O. Box 1962, United Arab Emirates; 4 Prosthodontics Department, Dr Ishrat-ul-Ebad Khan Institute of Oral Health Sciences, Dow University of Health Sciences, Karachi 74200, Pakistan; 5 Department of Restorative Dental Sciences, College of Dentistry, King Faisal University, Al-Ahsa 31982, Saudi Arabia; 6 Department of Prosthodontics, College of Dentistry, University of Science and Technology of Fujairah, Fujairah P.O. Box 2202, United Arab Emirates; 7 Department of Operative Dentistry and Endodontics, Altamash Institute of Dental Medicine, Karachi 75500, Pakistan

**Keywords:** endodontics, root canal treatment, cleaning, Er:YAG laser, smear layer

## Abstract

*Background and Objectives:* Smear layer forms during cleaning and shaping can obstruct the entry of both irrigant and sealant into the dentinal tubules, resulting in the accumulation of the bacteria and their byproducts. To ensure effective adhesion and better periapical healing, it is strongly advised to remove the smear layer before proceeding with root canal obturation. This study was designed to compare the efficiency of laser-activated irrigation (LAI) in removing the smear layer and debriding the most apical third of the root canal. *Materials and Methods:* Sixty-five extracted human teeth with straight single roots were randomly and equally divided into four laser and one control groups. Root canals in all samples were shaped using prime size TruNatomy rotary files. During preparation, each canal was irrigated with 3 mL of 3% NaOCl and 3 mL of 17% EDTA alternately, followed by the irrigation with 10 mL of distilled water to avoid the prolonged effect of EDTA and NaOCl solutions. Final irrigation of 5 mL of 17% EDTA of the root canal was done to eliminate the smear layer and was subsequently activated by an endodontic ultrasonic tip for 20 s three times (control group), a flat-end laser tip (test groups 1 and 3) or a taper-end laser tip (test groups 2 and 4) for two cycles. The time of each cycle activation was 10 s (groups 1 and 2) or 20 s (groups 3 and 4) in which the Er:YAG laser of 2940 nm was used. The laser operating parameters were 15 Hz and 50 μs pulse duration. The samples were then split longitudinally and subjected to scanning electron microscopy (SEM). *Results:* The remaining smear layer at the apical part of the root canals was statistically significant between the control group and the laser groups 1 (*p* = 0.040) and 2 (*p* = 0.000). Within the laser groups, the exposed tubules count was greater in the laser with the flat tip as compared with the tapered tip (Laser 1 > Laser 2 and Laser 3 > Laser 4). Finally, no significant differences in the count of debris between the laser groups and control group were observed, except for laser 4 (*p* < 0.05), which had the highest count of debris. *Conclusion:* LAI to remove debris and smear layer at the apical third of the root canal is inferior to the current ultrasonic technique. However, when using the Er:YAG LAI, it is recommended to use a flat tip design for 10 s for two cycles to ensure maximum debridement of the apical dentin surface.

## 1. Introduction

One of the crucial steps in root canal therapy that decide the fate of the treatment is the cleaning and shaping procedure [1,2]. In an ideal circumstance, the cleaning and shaping procedure is expected to remove most of the bacteria in the root canal; however, studies have shown that even the most thorough mechanical procedure is unable to remove organic residues and deeply located bacteria in the dentinal tubules [3,4,5]. This is further explained by the presence of anatomical complexities of the root canal such as fins, extensions, isthmus, accessory canals, or apical deltas that may prevent adequate cleaning by mechanical means only [6,7,8]. As a result, chemical agents such as sodium hypochlorite (NaOCl) and ethylenediamine tetraacetic acid (EDTA) are used to remove necrotic pulp tissue and debris and wipe away any microorganisms that mechanical instruments cannot remove and are strongly suggested during and immediately after the mechanical preparation of the root canal system [9]. 

Irrigation can lessen instrument-to-dentin friction. It can also help avoid the complicated and soft tissue packing in the apical root canal because some components in the periapical area may be infected [10,11]. NaOCl has the ability of tissue dissolution and antimicrobial activity; therefore, using it is of absolute importance [11]. While EDTA is considered high-priced, it may be worthwhile to use because of its capability in removing the smear layer. These solutions must cover the root canal walls completely to provide the best therapy. However, due to the complicated anatomies of the root apex, vapor lock phenomena occur in the apical third of the canal, which prevents static irrigation from soaking the whole root canal wall surface [12]. Consequently, the processes of agitation/activation are vital to allow these irrigating solutions to cover the root canal walls entirely.

Root canal irrigation with laser-activated irrigation (LAI) is a common technique for automatic agitation and activation of irrigants. For the LAI process, lasers of the erbium family, such as Er. Cr, YSGG, and Er:YAG are frequently employed. Water and NaOCl are good absorbers of the wavelengths utilized to begin at the mid-infrared region (2780–2940 nm) [13]. Frequencies of 1 to 100 Hz and pulse intensities of 5 to 1000 mJ are used in these lasers. Cavitation is an essential consideration in LAI because it causes the irrigant to flow in several directions and at varying lengths. To better understand how LAI works, researchers have turned to high-speed imagery [14,15]. This picture depicts the early formation and bursting of a massive vapor bubble at the fiber tip due to the irrigant’s quick heating [16]. It is determined by the laser’s output energy, wavelength, pulse duration, and irrigant absorption that the laser-generated bubble is large or small. Laser-induced bubble collapse causes fluid absorption from the coronal and apical body portions and the bubble centre encompasses all the lateral canals. Several factors, such as the temperature of the LAI and the laser tip design, have a critical effect on the cavitation process.

Several final irrigation protocols were described to improve the apical smear layer removal. Some are agitation using hand files, gutta-percha cones, or sonic and ultrasonic devices [15]. Using lasers to activate root canal irrigants was recently suggested [14,15,16,17]. Many laser types and protocols using Nd:YAG, Er:YAG, and laser diode have been introduced to increase the efficacy of irrigation solutions [15,18,19]. However, there is no consensus over the best laser irrigation protocol for removing the smear layer; therefore, this study was designed to see the efficacy of smear layer removal at the apical one-third of the root using different protocols of erbium-doped yttrium aluminium garnet (Er:YAG) laser.

## 2. Materials and Methods

### 2.1. Sample Selection

After obtaining the approval from the UDHS ethical committee (REC-20-03-23-02-S), sixty-five human extracted teeth were used in this study. The inclusion criteria were single-rooted intact mature teeth with straight canals without caries, cracks, and fractures and with no abnormal anatomy or previous root canal treatment. Teeth were kept for 48 h in 2.5% NaOCl to remove organic debris. Then, the external root surfaces were scaled with an ultrasonic scaler to remove any calculus or soft tissue washed with distilled water, and stored in saline until they were used.

The teeth were later decoronated by cutting the coronal part to standardize the length to 15 mm. Radiographs were taken from each tooth’s mesial and distal aspects to ensure that the apices were closed; the root apex was sealed with sticky wax.

### 2.2. Root Canal Preparation and Activation Procedures

After access cavity preparation, root canals were prepared using TruNatomy prime shaping files to form root canal systems into a constantly tapering preparation with the most peri-cervical dentin preservation possible.

The canals were irrigated with 3 mL of 3% NaOCl and 3 mL of 17% EDTA alternately without activation. Subsequently, every root canal was irrigated with 10mL of distilled water to avoid the prolonged effect of EDTA and NaOCl solutions. Final irrigation of 5 mL of 17% EDTA of the root canal was done to eliminate the smear layer. Then teeth were randomly divided into four laser groups and one control group (n = 13).

The final EDTA irrigants in all laser groups were activated using an Er:YAG laser of 2940 nm (Fidelis, Fotona, Ljubljana, Slovenia) following the free-running emission mode, and with a fiber-optic endodontic handpiece at panel settings of 1 W, 20 Hz, and 50 mJ; each using a different tip design and time duration.

Laser 1 group = Final irrigant was activated by Preciso/flat laser tip (Fidelis, Fotona, Ljubljana, Slovenia) for 10 s.

Laser 2 group = Final irrigant was activated by Xplus tapered laser tip (Fidelis, Fotona, Ljubljana, Slovenia) for 10 s.

Laser 3 group = Final irrigant was activated by Preciso/flat laser tip (Fidelis, Fotona, Ljubljana, Slovenia) for 20 s.

Laser 4 group = Final irrigant was activated by Xplus tapered laser tip (Fidelis, Fotona, Ljubljana, Slovenia) for 20 s.

Control group = The final EDTA irrigants were activated using an ultrasonic tip (IrriS-afe™, Acteon, Merignac, France) for a total time of 60 s (20 s × 3 cycles).

The laser tip was placed 12 mm away from the apex and was kept stationary with no advance into the canal in all laser samples. Irrigation with saline was achieved between every two cycles.

### 2.3. Temperature Measurements

The temperature variations on the external root surface of five teeth in each laser group (for a total of twenty teeth) were monitored using an infrared temperature sensor to identify any thermal side effects and establish the safety of the specified laser settings (Shenzhen Ju-maoyuan Science and Technology Co., BENETECH, Shenzhen, China). The sensor indicator was directed to the tooth surface at three coronal, middle, third, and apical pre-marked points. The marks were placed at 2, 8, and 12 mm far from the apex. Temperature variations were measured at each point during the irradiation technique, and the results were recorded.

### 2.4. Sample Fixation and Preparation

**Figure 1:**
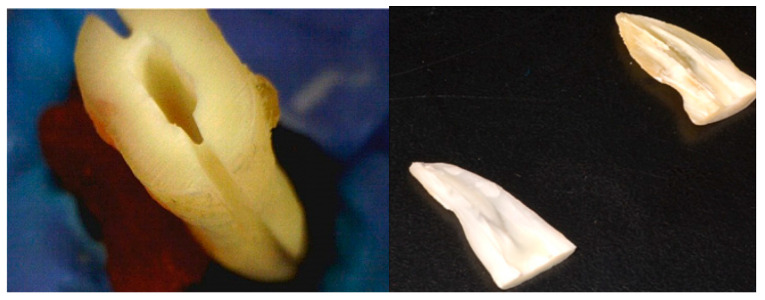
Longitudinal grooves were made on mesial and distal surfaces of the root using a diamond disk.

After irrigation and dryness of the sample, longitudinal grooves were made on their mesial and distal surfaces using a diamond disk (Figure 1) without perforating the canals. Sterile TruNatomy paper points in the prime size were placed into the canals after the biomechanical preparation to prevent canal contamination with dentinal chips during the root separation. A chisel splits the tooth into two parts. Samples were fixed by soaking each in formaldehyde for one hour and then washing the sample with buffered saline. Then samples were soaked in 50, 70, 90, and 100% ethyl alcohol for 10 min each, rinsed two times in buffered saline, and then allowed to air-dry for 72 h.

### 2.5. Scanning Electron Microscopy

Samples were coated with gold and palladium in a vacuum to a thickness of 20 nm and mounted on aluminum stubs. Then the entire area was examined 2 mm from the apex under SEM (SEM 5600, JEOL Ltd., Tokyo, Japan); two SEM images at 2000× magnification were recorded at a fixed distance of 2 mm from the apical foramen at the midpoint for each sample. Images were then saved digitally in TIFF format. The SEM images were assessed to determine the effectiveness of smear layer removal following the digital method described by Ciocca, et al. [20] in 2007 in which they used a totally automated computerized analysis technique to evaluate the open dentinal tubules and their surface area. In brief, the number of dentinal tubules, amounts of debris, and smear layer that remained at the apical third of the root canal of each specimen was scored separately using Matlab software. The scores of the remaining smear layer were classified into four groups as the following Table 1 shows.

### 2.6. Statistical Analysis

All the information was gathered, collated, and statistically analyzed in SPSS (Version 25.0., Armonk, NY, USA) while Microsoft Office Excel was used for data handling and graphical presentation. The Shapiro–Wilk test of normality was used to test the normality hypothesis of all quantitative variables for a different choice of appropriate parametric and non-parametric tests. The Mann–Whitney test was applied to compare the different laser groups to the control group for the remaining smear layer. The Dunnett *t*-test was used to compare all groups with the control group for the count of exposed dentinal tubules and the Bonferroni test was conducted to compare all laser groups to each other for the count of debris. The power of the study was 95% and a *p*-value < 0.05 was considered as statistically significant.

## 3. Results

**Figure 2:**
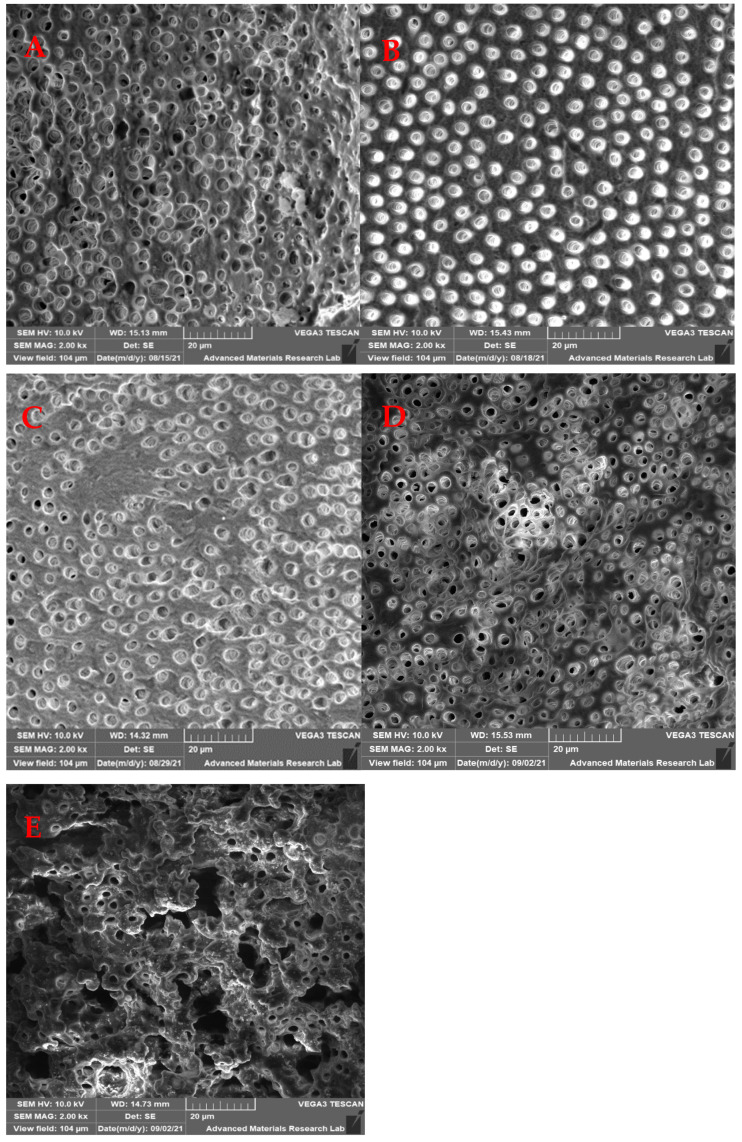
SEM photomicrograph (2000×) showing the remaining the smear layer at the apical third. (**A**) Ultrasonic group, (**B**) Laser 1 group, (**C**) Laser 2 group, (**D**) Laser 3 group, and (**E**) Laser 4 group.

**Figure 3:**
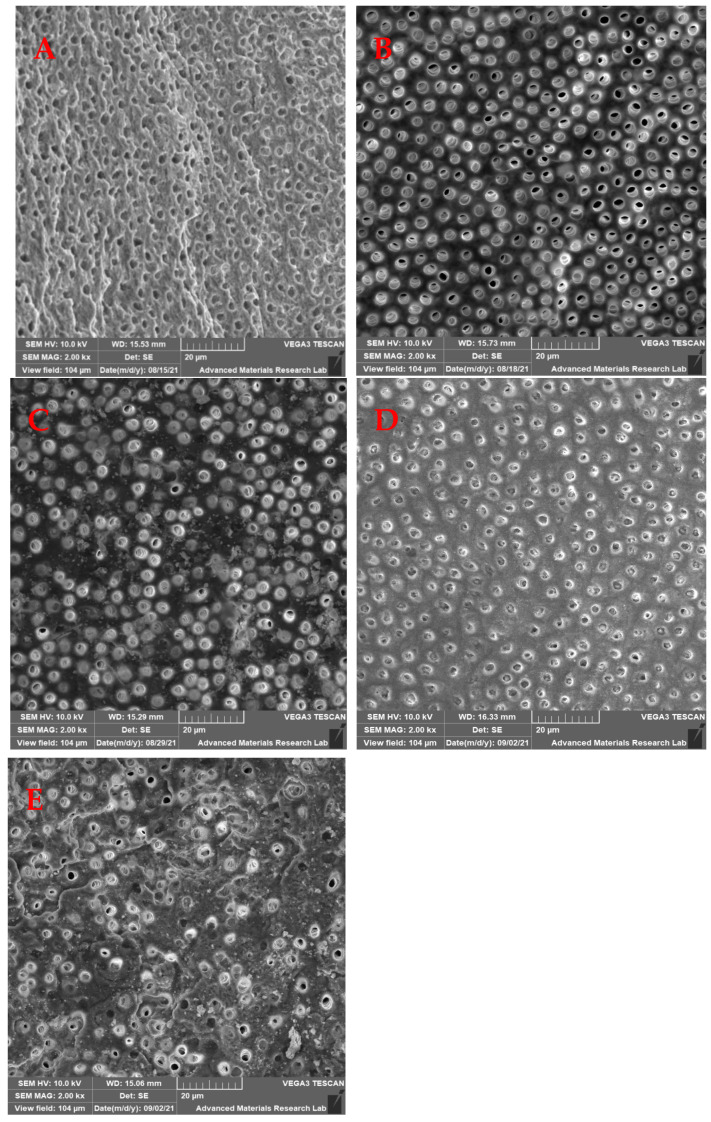
SEM photomicrograph (2000×) showing the exposed dentinal tubules at the apical third. (**A**) Ultrasonic group, (**B**) Laser 1 group, (**C**) Laser 2 group, and (**D**) Laser 3 group, (**E**) Laser 4 group.

**Figure 4:**
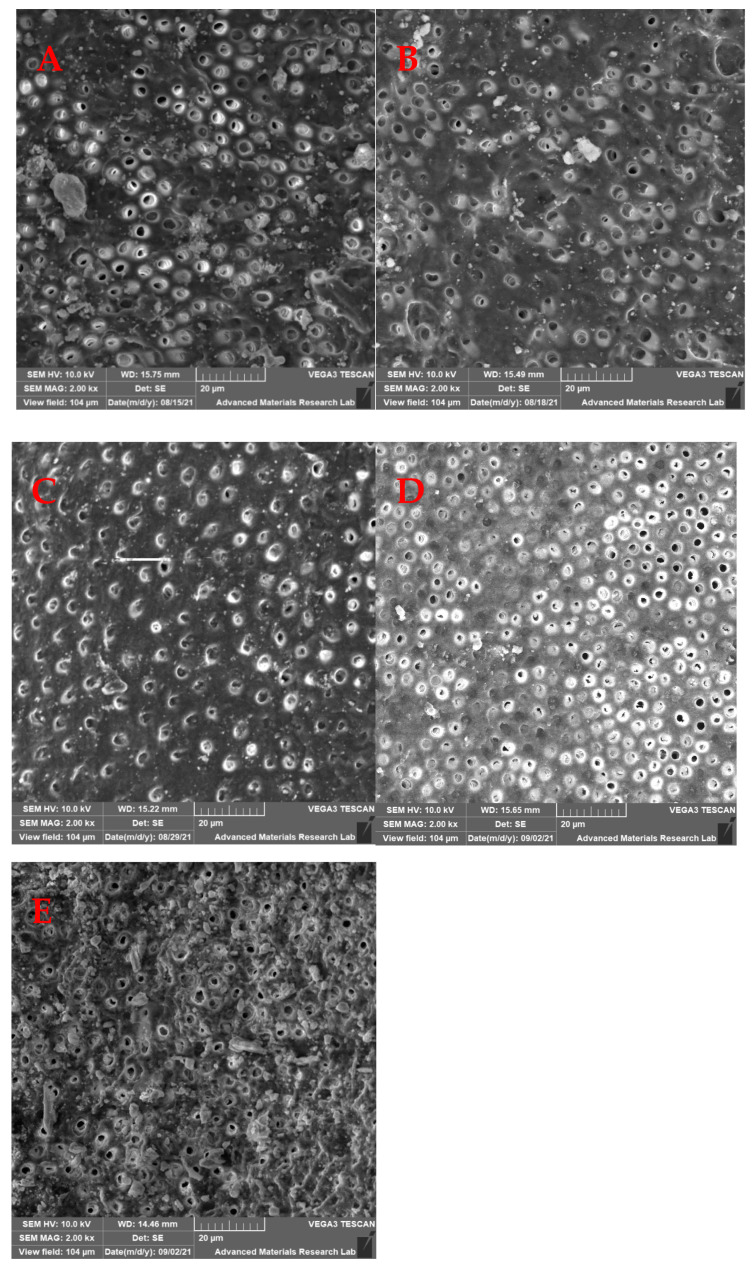
SEM photomicrograph (2000×) showing debris particles at the apical third. (**A**) Ultrasonic group, (**B**) Laser 1 group, (**C**) Laser 2 group, (**D**) Laser 3 group, and (**E**) Laser 4 group.

The mean values of the remaining smear layer, count of exposed dentinal tubules, and count of debris for the different groups were calculated using SEM images, and compared and analyzed as shown in Figure 2, Figure 3 and Figure 4.

### 3.1. Remaining Smear Layer Area

**Figure 5:**
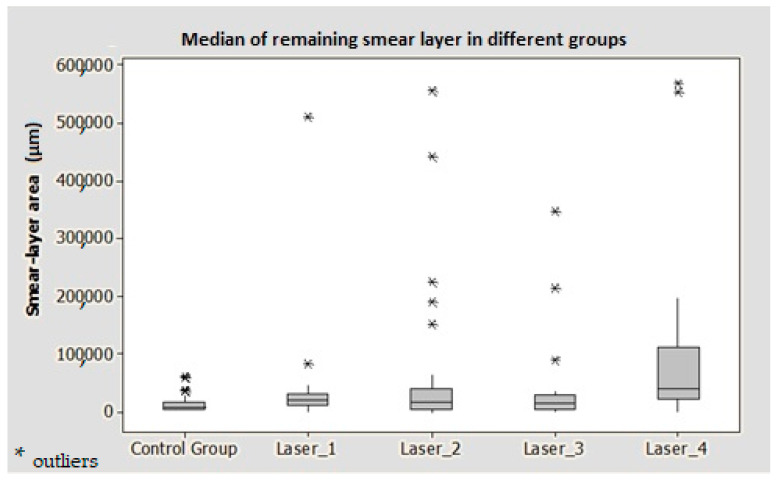
Box plot showing the comparison of the remaining smear layer between control and laser groups.

The median of the remaining smear layer score for all groups was 1 (<25% of the total area). However, the best smear layer removal was observed in the ultrasonic (control) group, followed by group 3, group 2, group 1 and group 4, respectively (Figure 5).

The remaining smear layer at the apical part of the root canals was statistically significant between the control group and the laser groups 1 (*p* = 0.040) and 4 (*p* < 0.001) (Table 2).

### 3.2. Count of Exposed Dentinal Tubules

**Figure 6:**
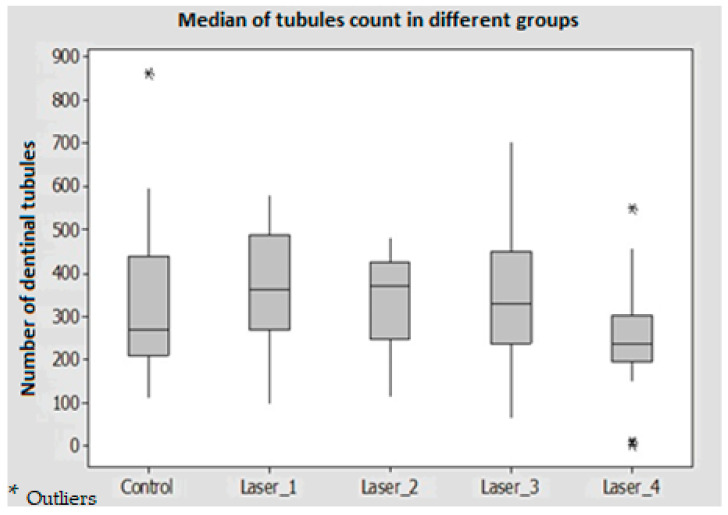
Comparison of exposed dentinal tubules count between the control and laser groups.

The results showed that there were no significant differences in all test groups compared to the control group (*p* > 0.05) (Table 2). Within the laser groups, the exposed tubules count was greater in the laser with the flat tip as compared with the tapered tip for 20 s (Laser 3 > Laser 4) while the opposite was observed when time activation time was reduced to 10 s (Laser 2 > Laser 1) (Figure 6).

### 3.3. Count of Debris

The results showed that there were no significant differences in the count of debris between the laser groups when compared to the control group, except for laser 4 (*p* < 0.05) which had the highest count of debris (Table 2).

The analysis of the effect of laser tip design in combination with the duration of laser application showed that there was a significant difference between Laser 1 and Laser 3, Laser 1 and Laser 4, and Laser 2 and Laser 4 (*p* < 0.01) (Table 3).

### 3.4. Temperature Changes

In our study the temperature changes on the outer root surface during activation, induced by an Er:YAG laser while applying 17% EDTA solution, was measured on the outer root surface. The irrigant was engaged for 10 and 20 s using 1 W, 20 Hz, 50 mJ, and a very short pulse length with a flat and tapered tip, resulting in a temperature change of no more than 4 °C. The average temperature range was (23.2–27.1 °C). 

## 4. Discussion

The present study aimed to evaluate the effectiveness of an Er:YAG laser protocol used to activate EDTA using different shapes of laser tips (flat and tapered) for different periods (10 and 20 s) for two cycles when and to compare it with the current common ultrasonic activation protocol. Three different parameters were analyzed using SEM images, these included the smear layer removal, count of exposed tubules, and count of remaining debris at the apical third.

The overall result of the current study shows that the least effective laser group was observed for Laser 4, where LAI was applied using a tapered fiber tip for 20 s. Both smear layer removal and debris removal were significantly lower than in the control group (PUI); this again can be interpreted by the model of the photoacoustic streaming that results from using that shape of fiber tip for that specific time. Based on this interpretation, other factors may play a role in the photoacoustic streaming model and the outcome of LAI. Some of the factors to be considered are the size, shape, and length of the root canal.

De Groot, et al. [21] studied three alternative irrigation methods for debriding a conventional root canal model filled with artificially generated dentin debris. Sodium hypochlorite (2%) was used as an irrigant for syringe irrigation, PUI, and LAI. With a 280 m flat tip and a reduction factor of 0.36 (effective fluence of 146 mJ mm^2^), an Er:YAG laser (Key 2, KaVo, Dental GmbH, Biberach, Germany) was utilized at 100 mJ, 15 Hz, with a calibration of the optical fiber resulting in a reduction factor of 0.36 (effective fluence of 146 mJ mm^2^). In the apical third of the root canal, the tip was placed 1 mm short of the working length and slowly pushed up and down 4 mm. The entire irrigation duration was 50 s, with a 20 s activation time. When triggered for 20 s, laser-activated irrigation (LAI) was substantially more successful in eliminating dentin debris from the apical region of the root canal than irrigation PUI or hand irrigation. When comparing our study with De Groot et al. [21], we found that in our study LAI was not better than PUI. However, LAI was almost comparable to PUI for the three laser groups (groups 1, 2, and 3). This can be interpreted by the different laser energy and power, which influences the bubble formation and size [14,15,22]. As we know that shorter pulses, in the range of a few microseconds, cause the bubble to start forming faster. Additionally, shorter pulses result in higher peak power [14,22]. The different locations of the laser tip between our study and De Groot’s study may also contribute to the difference in the final outcome of the LAI. In our study, the laser tip positioned was stationary at 12 mm from the apex without movement.

A study was done by De Moor et al. [23] in 2010 that compared the efficacy of LAI in removing debris in RCS to the conventional needle irrigation technique and passive ultrasonic irrigation (PUI) using Irrisafe (Satelec, Acteon group, Merignac, France) for only 20 s at a frequency of 30 kHz and a displacement amplitude of approximately 30 mm. Additionally, an Erbium Chromium: Yttrium Scandium Gallium Garnet (Er, Cr: YSGG) laser with a 200 m flat tip (Z2, Endolase Tip, Biolase) was used to activate 2.5% NaOCl four times for 5 s at 75 mJ, 20 Hz, 1.5 W, positioned stationary at 5 mm from the apex, and prepared at ISO #40. LAI produced statistically significantly less debris than PUL and (CI) (*p* = 0.005) after 20 s. In the current study, the efficiency of debris removal using LAI was comparable to PUI when using the flat tip (groups 1 and 3). This disagreement in the findings between this study and the study from De Groot et al. [18] may be interpreted by using different settings of LAI, the different diameters and locations of the laser tip, and, maybe, the difference in the size of the prepared root canal, as rotary files were used to prepare the root canals in our study.

In our study, the temperature changes on the outer root surface during activation induced by an Er:YAG laser utilizing 17% EDTA solution were measured on the outer root surface. The results showed that the temperature change was not more than 4 °C, and that the average temperature range was 23.2–27.1 °C. Kreisler et al. [24] reported that an increase in temperature by 5.5 °C even for one minute could cause irreversible pulpitis. Similarly, a temperature increase of 10 °C for one minute could initiate alveolar bone necrosis [25]. Nevertheless, a temperature increase of up to 7 °C is considered safe for periodontal tissue [26]. Based on these results, the changes in temperature produced by laser-induced cavitation, following the current settings and shape tips, are generally safe and applicable in clinics. This result is also consistent with previous studies where the Er:YAG laser could remove the smear layer from root canal walls [21,27,28,29,30].

This is an in vitro study where the experiment’s conditions may not represent the real clinical conditions; therefore, the results should be extrapolated carefully. First, the mechanism of LAI action contains generating waves and bubbles that traveled into the irrigant and is followed by the implosion of irrigant bubbles. The interaction between the original waves and the waves that are reflected from the canal walls may differ according to the size of the prepared root canal. The root canals in this study were prepared to a standard volume using a rotary file system. This means, that the LAI results found in this study may differ if applied to other canals that were prepared using different files. Second, the change in the distance between the laser tip and the apical third may also result in different findings for the same reason. The distance of the 12 mm applied in this study may not be applicable for all teeth that need root canal treatment. Lastly, the results might differ with other rotary file systems; therefore, further studies are desirable to thoroughly evaluate the efficiency of laser-activated irrigation (LAI) while keeping these limitations in mind.

## 5. Conclusions

LAI to remove debris and smear layer at the apical third of the root canal is inferior to the current ultrasonic technique. However, when using the Er:YAG LAI, it is recommended to use a flat tip design for 10 s for two cycles to ensure maximum debridement of apical dentin surface.

## Figures and Tables

**Table 1:** Score and classification of the remaining smear layer.

Score	Classification of Remaining Smear Layer Area
Score 1	The smear layer covers < 25% of the total area
Score 2	The smear layer covers 25–49% of the total area
Score 3	The smear layer covers 50–74% of the total area
Score 4	The smear layer covers > 75% of the total area

**Table 2:** Comparison of all laser groups to the control group in terms of remaining smear layer area, exposed dentinal tubules, and count of debris.

Groups		Remaining Smear Layer	Exposed Dentinal Tubules	Count of Debris
	**Laser 1**	0.040 *	0.779	0.607
**Control Group (Ultrasonic)**	**Laser 2**	0.431	0.996	0.999
	**Laser 3**	0.634	0.988	0.232
	**Laser 4**	0.000 *	0.067	0.001 *

(*) statistically significant *p*-value.

**Table 3:** Bonferroni method for multiple comparisons of all groups—count of debris, (*) significant result.

Groups		*p*-Value
**Laser 1** **(Flat × 10 s)**	**Laser 2** (Taper × 10 s)	1.000
**Laser 1** **(Flat × 10 s)**	**Laser 3** (Flat × 20 s)	0.041 *
**Laser 1** **(Flat × 10 s)**	**Laser 4** (Taper × 20 s)	0.000 *
**Laser 2** **(Taper × 10 s)**	**Laser 3** (Flat × 20 s)	0.967
**Laser 2** **(Taper × 10 s)**	**Laser 4** (Taper × 20 s)	0.005 *
**Laser 3** **(Flat × 20 s)**	**Laser 4** (Taper × 20 s)	0.627

(*) statistically significant.

## Data Availability

Not applicable.
